# Retraction: Bidirectional rotating flow of nanofluid over a variable thickened stretching sheet with non-Fourier’s heat flux and non-Fick’s mass flux theory

**DOI:** 10.1371/journal.pone.0329026

**Published:** 2025-07-29

**Authors:** The PLOS One Editors

The *PLOS One* Editors retract this article [1] due to concerns about potential manipulation of the publication process and adherence to the journal’s first publication criterion. These concerns call into question the validity and provenance of the reported results. We regret that the issues were not identified prior to the article’s publication.

MI did not agree with the retraction. FM, MR, EREZ, MOS, and MIK either did not respond directly or could not be reached.
